# High rates of viral co-detection in outpatient children with acute respiratory infections after the easing of preventive measures against Covid-19 in Germany

**DOI:** 10.1186/s12879-026-13125-9

**Published:** 2026-03-24

**Authors:** André Haufschild, Patricia Niekler, Johanna Sack, Benedikt Weissbrich, Kerstin Knies, Christoph Härtel, Andrea Streng, Lars Dölken, Johannes G. Liese, Geraldine Engels

**Affiliations:** 1 Department of Pediatrics, University Hospital Wuerzburg, Josef-Schneider-Str. 2, D-97080 Wuerzburg, Germany; 2 Institute for Virology and Immunobiology, University of Wuerzburg, Wuerzburg, Germany; 3 Institute for Virology, Medizinische Hochschule Hannover, Hannover, Germany

**Keywords:** Acute respiratory infections, Respiratory viruses, Co-detection, Children, Outpatient setting

## Abstract

**Background:**

Following the easing of preventive, non-pharmaceutical COVID-19 interventions (NPIs) during the late phase of the pandemic, acute respiratory infections (ARI) in children reemerged. We investigated the viral etiology, seasonality and clinical characteristics of children with ARI treated in primary care practices during this period.

**Methods:**

We conducted a prospective, observational study on children ≤ 14 years of age, presenting with acute upper (URTI) or lower (LRTI) ARI in five pediatric primary care practices in Wuerzburg, Germany, from 10/2021 to 05/2022. A maximum of eight children per practice were included on one predefined day per week. Oropharyngeal swabs were analyzed using (multiplex) PCR for 18 viral pathogens.

**Results:**

A total of 521 children (median age 3.3 years; IQR 1.6–5.5; 52% male) with ARI were enrolled (28% LRTI). At least one virus was detected in 85%, with human rhinovirus (HRV) (30%), Respiratory Syncytial Virus (RSV) (22%), and SARS-CoV-2 (16%) being the most frequent viruses. Co-detection (≥ 2 viruses) occurred in 156 (30%) patients, were most frequent in children < 2 years of age (38% of 164), and their rate decreased with increasing age (*p* = 0.012). Bocavirus (88% of 56), adenovirus (80% of 48) and endemic coronaviruses (70% of 42) showed the highest proportion of co-detection. RSV and human metapneumovirus (hMPV) were predominant in LRTI, whereas HRV and SARS-CoV-2 were more prevalent in URTI. The LRTI rate was 32% of 288 mono-detections and 23% of 156 co-detection.

**Conclusions:**

During fall/winter 2021/2022, viral co-detection were common, possibly as a consequence of easing COVID-19-related NPIs, but were not associated with more severe clinical characteristics.

**Supplementary Information:**

The online version contains supplementary material available at 10.1186/s12879-026-13125-9.

## Introduction

During the early COVID-19 pandemic, the introduction of preventive, non-pharmaceutical COVID-19 interventions (NPIs) resulted in a strong decrease in respiratory virus circulation not only regarding SARS-CoV-2 but also of many other common respiratory viruses. Following the reduction in NPIs during the late phase of the pandemic, acute respiratory infections (ARI) in children have reappeared in many countries. Not only did the number of ARI increase, some of the viruses circulated outside their normal time and peaked unusually high [1–3]. For example, during 2020/2021 in Australia, the RSV peak was later and higher than usual [4]. However, other viruses, such as influenza, were less frequently detected in late fall/early winter 2021/2022 in many European countries, including Germany, despite easing of NPIs [5].

Although testing for respiratory pathogens has become more common during the COVID-19 pandemic, many studies have focused on hospitalized cohorts, which usually represent more severely ill patients or patients with underlying diseases [6–8]. Pediatric primary care practices, however, are often the primary contact point for the general pediatric population with ARI [9–11], but data on the etiology of ARI in outpatient settings are still limited.

In a previous study conducted by our group, on outpatient pediatric practices in Germany during the early COVID-19 pandemic (2020/21), we found continuous detection of rhino-, adeno- and endemic coronaviruses in children regardless of NPIs. RSV and influenza, however, were not detected when NPIs were present [12].

Yet, the epidemiological situation concerning the prevalence and circulation of respiratory viruses in outpatient children after the easing of NPIs was unknown and difficult to predict, whereas many of the national recommendations on preventive measures are based on the seasonal occurrence of these viruses. In particular, it remained unclear which respiratory viruses predominated in pediatric primary care settings after NPIs were lifted, how SARS-CoV-2 contributed to the outpatient ARI burden relative to established respiratory viruses and whether viral co-detections were common and clinically relevant in this population. As pediatric outpatient care represents the first point of contact for most children with acute respiratory infections, improved understanding of virus circulation in this setting is essential for early public health surveillance and for informing the timing and adaptation of preventive strategies. Therefore, we investigated the viral etiology, frequency of viral mono- and co-detections and associated clinical characteristics of children presenting with acute respiratory infections in pediatric primary care practices during the late phase of the COVID-19 pandemic (fall/winter 2021/2022), when non-pharmaceutical interventions were gradually eased in Germany.

## Methods

### Study setting and conduct

We performed a prospective, multicenter observational study using a study design identical to that of our previous investigation [12], from October 10th 2021 to May 25th 2022. Five (28%) of the 18 pediatric primary care practices in Wuerzburg (Germany) participated during this period. The city of Wuerzburg has approximately 130,227 inhabitants, including 15.500 children ≤ 16 years of age (31.12.2022) [13].

Children up to 14 years of age, presenting at one of the participating practices with symptoms of acute upper respiratory infection (URTI) (rhinitis, cough, and/or sore throat), or with acute lower respiratory tract infection (LRTI) (tachypnoea, hypoxia) were eligible, if these symptoms had appeared within the previous 14 days. Children with both URTI and LRTI symptoms were categorized as having LRTI. Exclusion criteria included prior enrollment in the study with ongoing infectious symptoms or a history of infection within the preceding 28 days.

A maximum of eight eligible patients were included in each primary care practice on one specific, predefined day per week, after parental informed consent. The practitioner collected an oropharyngeal swab and recorded the patient’s basic demographic and medical data using a questionnaire (Supplement 1).

### Viral diagnostics

Viral diagnostics using oropharyngeal swabs were performed at the Institute of Virology and Immunobiology, University of Wuerzburg, as previously described [12]. Oropharyngeal swabs were transported in a viral transport medium (COPAN ITALIA SPA, Brescia, Italy or biocomma, Guandong, China) and screened for viral pathogens using a commercial multiplex polymerase chain reaction (PCR; FTD Respiratory pathogens 21, Fast Track Diagnostics, Luxembourg). The FTD-21 test kit identified the following viral pathogens: influenza virus A and B, RSV, human parainfluenza virus 1–4, human coronaviruses NL63, OC43, HKU1 and 229E, hMPV, human bocavirus, adenovirus, HRV, enterovirus and parechovirus. SARS-CoV-2-RNA was identified by reverse transcription quantitative PCR. Cycle threshold values of ≥ 40 were considered negative. “Co-detection” was defined as the detection of two or more viral pathogens.

### Data analysis

All data were entered into IBM SPSS Statistics (version 29.0) for statistical analysis. Data were described as numbers and percentages, and medians with quartiles (Q1-Q3) to describe the interquartile range (IQR). Subgroups were compared using the Mann-Whitney U-test, Chi-squared test, and P-values < 0.05 were considered statistically significant. Binary logistic regression analysis was also performed to calculate the odds ratios and confidence intervals.

### Ethical considerations

The Ethics Committee of the Medical Faculty at the University of Wuerzburg approved this study (183/20-sc). All parents/guardians provided written informed consent to participate in the study.

## Results

### Patient characteristics and clinical diagnoses

A total of 521 children (median age 3.3 years; IQR 1.6–5.5; 52% males) with ARI were recruited. Sixty-two (12%) patients had an underlying disease, with recurrent obstructive bronchitis being the most frequent (41/521; 8%) (Table 1). Of the 521 study participants, 375 (72%) presented with symptoms of URTI and 146 (28%) with symptoms of LRTI. Rhinitis, cough, and fever were the most common symptoms in both groups, but cough was significantly more frequent among children with LRTI than URTI (99% vs. 75%, *p* < 0.001). Of the participants with LRTI, 117/146 (80%) were diagnosed with bronchitis/bronchiolitis, 13/146 (9%) with pneumonia and 16/146 (11%) with undifferentiated LRTI. Four of the 521 patients (0.8%) required immediate hospitalization.

**Table 1:** Patient characteristics and clinical diagnoses of outpatient children

PATIENT CHARACTERISTICS	ALL PATIENTS n = 521	URTI n = 375	LRTI n = 146	P-VALUE
Male (n, %)	273 (52)	192 (51)	81 (55)	0.380
Age in years (median; IQR)	3.3 (1.6–5.5)	3.5 (0.3–5.9)	2.8 (1–5)	0.704
Hospitalization (n, %)	4 (0.8)	2 (0.5)	2 (1.4)	0.326
Underlying disease (n, %) -Recurrent obstructive bronchitis (n, %)	62 (12) 41 (8)	36 (10) 20 (5)	26 (18) 21 (14)	0.160 < **0.001**

Data was collected in five pediatric primary care practices in Wuerzburg from October 2021 - May 2022. Upper (URTI), lower (LRTI) respiratory infection, n=number

### Virus detections

#### Overall

In 444/521 (85%) of all study participants, at least one virus species was detected (overall 638 virus detections). Co-detections (≥ 2 viruses) occurred in 156/521 children (30%). HRV was the most frequently detected virus (155/521; 30%), followed by RSV (114/521; 22%) and SARS-CoV-2 (82/521; 16%) (Table 2).

**Table 2:** Comparison of viral pathogens detected in 521 outpatient children

Patient with VIRUS detected*	All Patients *n* = 521	URTI (*n* = 375)	LRTI (*n* = 146)	*P*-VALUE
HRV	155/521 (30%)	123/375 (33%)	32/146 (22%)	**0.015**
RSV	114/521 (22%)	72/375 (19%)	42/146 (29%)	**0.018**
SARS-CoV-2	82/521 (16%)	69/375 (18%)	13/146 (9%)	**0.008**
hMPV	74/521 (14%)	45/375 (12%)	29/146 (20%)	**0.021**
hBoV	56/521 (11%)	38/375 (10%)	18/146 (12%)	0.467
AdV	48/521 (9%)	40/375 (11%)	8/146 (6%)	0.066
hCoV	42/521 (8%)	33/375 (9%)	9/146 (6%)	0.240
HPIV1-4	31/521 (6%)	23/375 (6%)	8/146 (6%)	0.777
HRV +/ or. EV	23/521 (4%)	17/375 (5%)	6/146 (4%)	0.833
Influenza virus	7/521 (1%)	3/375 (1%)	4/146 (3%)	0.084
hPeV	6/521 (1%)	3/375 (1%)	3/146 (2%)	0.228

*Multiple detections of viral pathogens per child possible

Data was collected in five pediatric primary care practices in Wuerzburg from October 2021 - May 2022. N=number, upper (URTI) or lower (LRTI) respiratory infection. HRV: human rhinoviruses; RSV: Respiratory Syncytial Virus; hMPV: human metapneumovirus; hBoV: human bocavirus; AdV: adenovirus; hCoV: human coronaviruses; HPIV 1–4: human parainfluenzaviruses 1–4; EV: enterovirus; hPeV: human parechovirus

#### Virus detections over time

Regarding viral circulation, RSV was detected mainly in October–December 2021, with the highest peak observed in November, affecting (60/96) 63% of all patients presenting with ARI in November. SARS-CoV-2 was detected from January–May 2022, and hMPV was detected mainly from January to May 2022, with a peak in March 2022. Influenza was detected at a low level from March 2022 to May 2022. HRV, adenovirus, endemic coronavirus, PIV and bocavirus were observed each month from October 2021 to May 2022.

#### Virus mono-detections

**Fig. 1:**
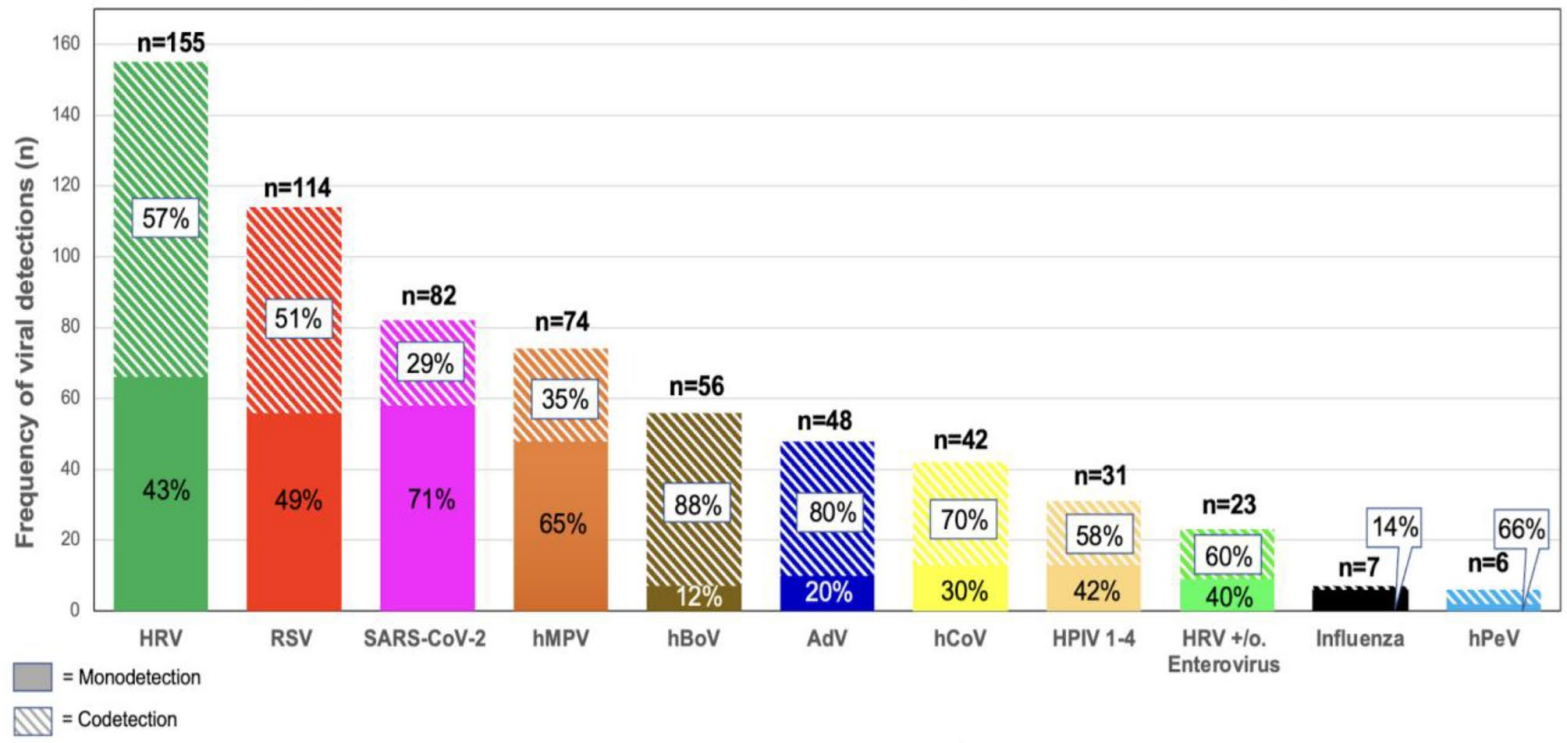
Viral detections (*n* = 638) in 521 children with acute respiratory infections stratified by mono- and co-detection. Children without viral detection were not included. HRV: human rhinovirus, RSV: respiratory syncytial virus, hMPV: human metapneumovirus, HPIV: human parainfluenza virus 1–4, AdV: adenovirus, EV: enterovirus, hBoV: human bocavirus, hPeV: human parechovirus, n=number

In 288/521 (55%) patients, only a single virus was detected (‘mono-detection’), whereas in 156/521 (30%) patients more than one virus was found. No virus was detected in the remaining 15%. In cases with a mono-detection, the three most frequently detected viruses were HRV with 23% (66/288), SARS-CoV-2 with 20% (58/288) and RSV with 19% (56/288) (Fig. 1).

#### Virus co-detection

The most common viruses identified in the 156 patients with co-detection were HRV (89/156; 57%), RSV (58/156; 37%) and bocavirus (49/156; 31%). The viruses with the highest rate of co-detection were bocaviruses (88% of 56), adenoviruses (80% of 48) and endemic coronaviruses (70% of 42) (Fig. 1).

**Fig. 2:**
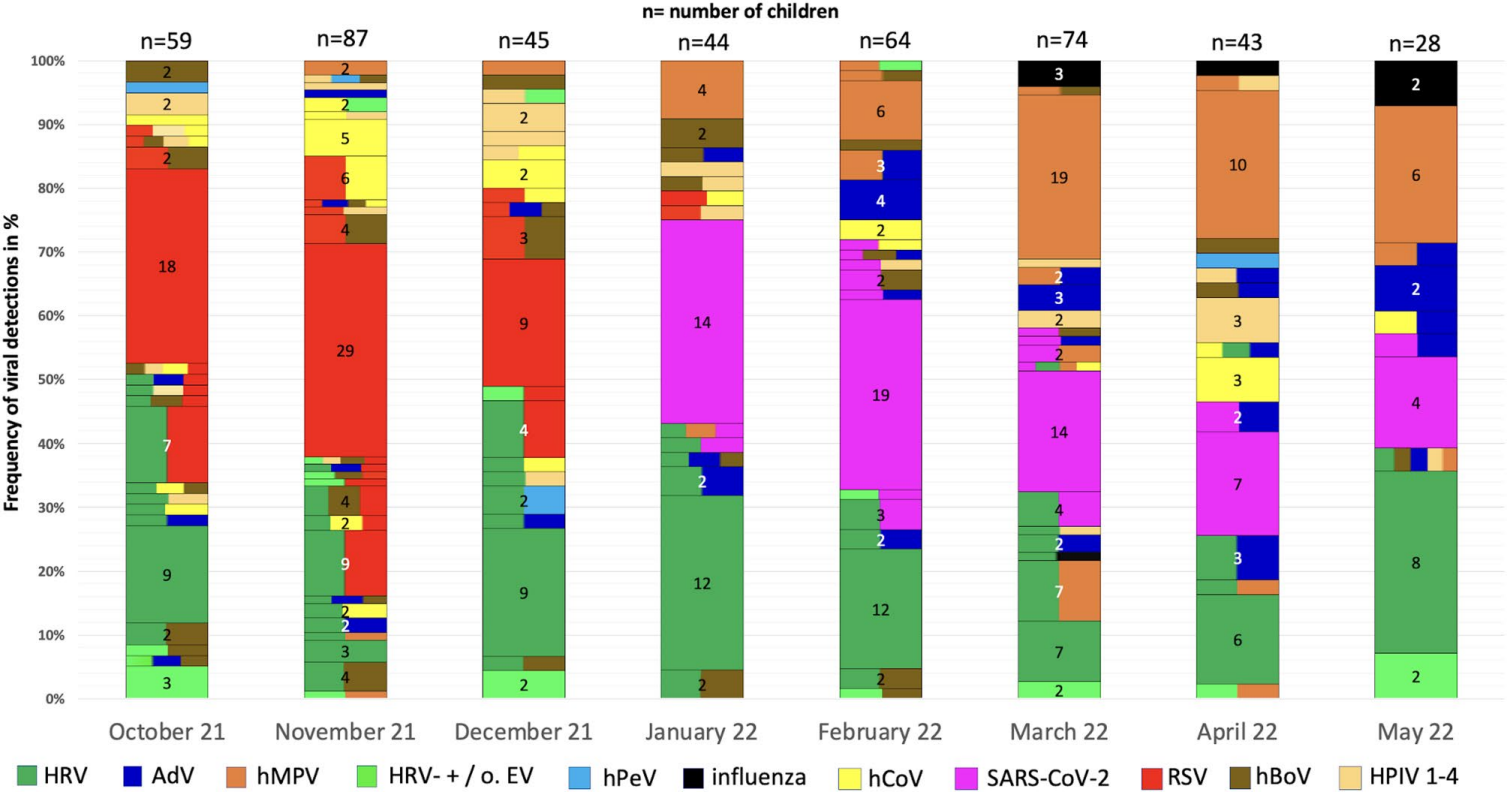
Overview of viral (co-)detections (*n* = 638) in 521 children with acute respiratory infections. Data was collected in five pediatric primary care practices from October 2021 - May 2022. n= number of patients with virus detection included in each month, children without viral detection were not included in the columns (Oct: *n* = 8, Nov: *n* = 9, Dec: *n* = 9, Jan: *n* = 44, Feb: *n* = 14, Mar: *n* = 11, Apr: 11, May: 28). Numbers within the bars indicate the absolute frequency of pathogen detection. If two or more colors appear in a single bar, it indicates the detection of the respective viruses simultaneously. HRV: human rhinovirus, RSV: respiratory syncytial virus, hMPV: human metapneumovirus, HPIV: human parainfluenza virus 1–4, AdV: adenovirus, EV: enterovirus, hBoV: human bocavirus, hPeV: human parechovirus, n=number

In children with co-detection, certain viral combinations appeared more frequently than others: i.e. HRV was co-detected in 57% (89/155) cases among all children who tested positive for HRV, mainly in combination with RSV (31/89; 35%), followed by bocavirus (21/89; 24%) and adenovirus (19/89; 21%). RSV was co-detected in 51% (58/114) of cases, mainly in combination with HRV (31/58; 53%), followed by bocavirus (21/58; 36%) and endemic coronavirus OC43 (13/58; 22%). SARS-CoV-2 was co-detected in 29% (24/82) of cases, mainly in combination with HRV in 10 of 24 (42%) cases, followed by adenovirus (6/24; 25%), bocavirus and hMPV in 4 of 24 (17%) cases. hMPV occurred as a co-detection in 35% (26/74) of cases, mainly in combination with HRV in 12/26 (46%) cases, followed by adenovirus in 7/26 (27%) cases and SARS-CoV-2 in 4/26 (15%) cases (Fig. 2).

Co-detection were found in children < 2 years of age (YOA) at a rate of 38%, in children 2–4 YOA at 32%, in the 5–10 YOA at 20% and in 11% of the 11–14 year old participants (*p* = 0.012) (Table 3).

**Table 3:** Comparison of number of viruses detected simultaneously in children with upper or lower respiratory infection

PATIENT CHARACTERISTICS	N	NO VIRUS DETECTION	1 VIRUS DETECTION	> 1 VIRUS DETECTION
0-1 Years	164	27 (17%)	75 (46%)	62 (38%)
2–4 Years	203	25 (12%)	114 (56%)	64 (32%)
5–11 Years	145	24 (17%)	92 (63%)	29 (20%)
12–14 Years	9	1 (11%)	7 (78%)	1 (11%)

Data was collected in five pediatric primary care practices from October 2021 - May 2022. N=number

#### Virus mono- and co-detection by severity (URTI/LRTI)

Overall, children with co-detection had a lower rate of LRTI (36/156; 23%) than children with mono-detections (92/288; 32%). Looking at RSV in more detail, children with RSV mono-detection were more often diagnosed with LRTI (29/56; 52%) than those with RSV and another virus (13/58; 24%).

#### Virus detection in children with URTI

**Fig. 3:**
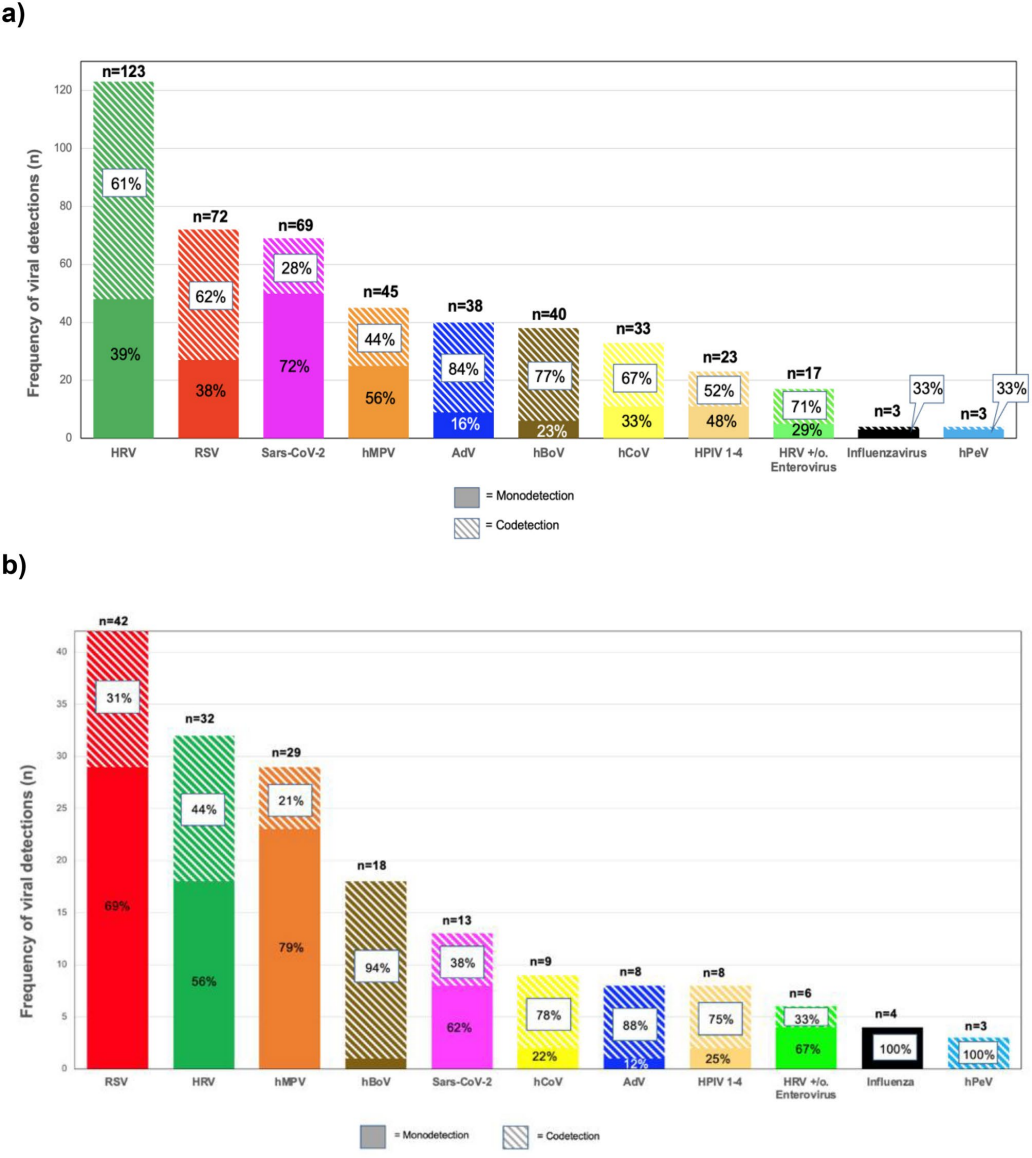
Viral detections stratified by mono- and co-detection in URTI and LRTI. (**a**) shows 375 children with upper respiratory infection (*n* = 466) and (**b**) 146 children with lower respiratory infections (*n* = 167). Children without viral detection were not included. URTI: Upper respiratory infections, LRTI: lower respiratory infection, HRV: human rhinovirus, RSV: respiratory syncytial virus, hMPV: human metapneumovirus, HPIV: human parainfluenza virus 1–4, AdV: adenovirus, EV: enterovirus, hBoV: human bocavirus, hPeV: human parechovirus, n=number

Among the 521 participants, 375 were diagnosed with URTI. In 316 of the 375 (84%) infections, at least one virus species was detected, with HRV (123/375; 33%) being the most frequently detected virus, followed by RSV (72/375; 19%) and SARS-CoV-2 (69/375; 18%) (Fig. 3a). In 120 of 375 (32%) participants, more than one virus was detected.

#### Virus detection in children with LRTI

LRTIs were diagnosed in 146 of all 521 study participants. In 128 of 146 (88%) participants with LRTI, at least one virus species was detected. In 42 of 146 (29%) LRTI cases, RSV was detected, followed by HRV (22%; 32/146) and hMPV (20%; 29/146) (Fig. 3b). In 36 of 146 (25%) participants with LRTI, more than one virus was detected, whereas the rate of co-detection in children with URTI was 32% (*p* = 0.1).

#### Comparison of virus detection in children with URTI and LRTI

When comparing the occurrence of viruses in URTI and LRTI, RSV and hMPV were detected more frequently in LRTI (*p* = 0.018 for RSV and *p* = 0.021 for hMPV), whereas hRV and SARS-CoV-2 were more frequent in URTI (*p* = 0.015 for hRV and *p* = 0.008 for SARS-CoV-2) (Table 3).

#### Age and LRTI

Binary logistic regression was performed to assess the effect of age on the likelihood of a patient presenting with acute lower respiratory infection (LRTI). The overall model was statistically significant, X^2^ [2] = 33.392, *p* < 0.001, explaining 7.6% (Nagelkerke R^2^) of the variance in LRTI and correctly classifying 58.9% of the cases. Age was significantly associated with decreased odds of LRTI (OR = 0.845, 95% CI [0,790;0,0,903], *p* < 0.001).

## Discussion

In the present study, during the COVID-19 pandemic in fall/winter of 2021/2022, HRV, RSV and SARS-CoV-2 were the most frequently detected pathogens. Frequency data during the last 3 months of 2021 showed RSV, rhino- and endemic coronaviruses as the three most frequent pathogens, similar to data from the German nationwide physician-based sentinel surveillance system, consisting of approximately 590 primary care practices (24% RSV, 19% HRV and 14% endemic coronaviruses) [14]. Furthermore, the sentinel surveillance confirmed an increase in SARS-CoV-2 in the first months of 2022 to the 3rd most frequent pathogen in the observation period [15].

In Germany, the emergence of the SARS-CoV-2 Omicron variant in December 2021 led to widespread infections across the population. This included young and school-aged children who had previously been less affected than teenagers and adults [16]. At the same time, RSV resurged at higher levels compared to pre-pandemic times [17–23]. In our present cohort, RSV circulation ended by December 2021, while the first cases of SARS-CoV-2 emerged in January 2022. Furthermore, RSV and hMPV were significantly more commonly detected in children with lower respiratory infections (LRTI) compared to those with upper respiratory infections (URTI) (*p* = 0.018 for RSV and *p* = 0.021 for hMPV). Additionally, children under the age of two had higher rates of LRTI compared to older children. This age-related difference may be explained by epidemiological data indicating that primary RSV infection in early childhood, particularly during the first two years of life, is more likely to result in LRTI [24]. Whether the circulation of SARS-CoV-2 and RSV is mutually exclusive remains uncertain. Notably, hMPV was detected during the same months as SARS-CoV-2; however, co-detection of these two viruses was rare, suggesting limited simultaneous infection.

However, due to NPIs, young children under the age of four years in Germany have grown up in an environment with significantly less contact to respiratory viruses [16]. This lack of contact can lead to a so-called “immune debt”, possibly rendering the children more susceptible to multiple pathogens at the same time in the late pandemic and postpandemic era [25].

In our study, we showed that viral co-detection occurred in 30% of our cohort, which is higher than previously published data (25% [26]/ 21% [27]/ 18% [28]). HRV and RSV, the most frequently detected pathogens in our study, also appeared most frequently as co-detection. This observation aligns with the hypothesis that viruses well adapted to the upper respiratory tract, such as HRV and RSV, tend to circulate more persistently and are therefore more commonly detected, both as primary pathogens and in co-detection [29, 30].

The rate of co-detection declined with increasing age of the participants in our study, suggesting that young children in the first five years of life are exposed to multiple pathogens simultaneously or within a short interval-possibly for the first time-thereby potentially resulting in slower viral clearance. As we are not aware of a method to retrospectively assess which pathogen was the main infection/symptom-causing agent, we decided to use the term co-detection rather than co-infection. It is also important to note that not all co-detected viruses necessarily cause illness, as some may be present without contributing to the current disease symptoms.

Other authors have similarly observed an increase in viral co-detection following the lifting of non-pharmaceutical COVID-19 mitigation strategies [16]. The reasons for this observation remain speculative: as we and others have previously shown [12, 16, 31], certain viruses – such as rhino-, adeno- and endemic coronaviruses – circulated continuously throughout and after COVID-19 lockdowns. Furthermore, some studies have found that certain viruses, such as HRV, can be detected in asymptomatic or oligosymptomatic children, suggesting a non-causal relationship between detection of a virus and clinically relevant infection. However, in the first phase of the COVID-19 pandemic in May/June 2020, HRV were the only pathogens that still seemed to be associated with respiratory illness, mostly URTI [7]. Possible explanations include transmission through fomites and direct smear/contact. Consistent with this, in our cohort, HRV was detected in 61% of URTI cases, often alongside other viruses. It remains unclear whether HRV plays an active role in these infections [32].

Interestingly, our results showed that the simultaneous detection of multiple pathogens was not associated with more severe clinical outcomes such as LRTI. While data on this topic are conflicting-with some studies suggesting an association with increased disease severity [33] – it is important to note that these findings were primarily from hospitalized cohorts [28, 34]. In contrast, other studies reported findings consistent with our observations [35]. Data published before the COVID-19 pandemic also indicate that only certain viruses are likely to emerge and be detected in a host simultaneously, though it remains unclear whether this reflects true simultaneous infection or merely co-detection [36]. Taken together, the role of viral co-detection on clinical outcome remains controversial and difficult to compare, even within similar age groups and viruses assessed. It is important to understand that our study aims to provide an accurate picture of the epidemiological and clinical situation in pediatric outpatient settings. The inclusion of children with chronic underlying conditions was a deliberate design choice, as these patients constitute an integral part of routine pediatric outpatient care and frequently seek medical attention for respiratory infections. However, their inclusion may influence the observed epidemiological and clinical patterns and should be considered when interpreting the results, particularly with regard to generalizability to the broader pediatric population. In addition, certain risk factors such as a positive case index in the family or daycare attendance were not included in the survey.

Furthermore, although recruitment in each participating pediatric primary care practice was performed on a fixed weekday, different practices contributed data on different weekdays, resulting in patient inclusion across all working days of the week. Nevertheless, very short-term fluctuations in virus circulation within individual practices may not have been fully captured.

This study specifically captures a distinct transitional phase of the COVID-19 pandemic, during which non-pharmaceutical interventions were progressively eased. This period was characterized by rapid and atypical changes in respiratory virus circulation that are unlikely to be observed once population-level interventions are fully withdrawn. Although the data were collected in 2021–2022, the findings remain relevant, as they provide important insights into respiratory virus dynamics following abrupt changes in public health measures and help interpret current surveillance data and preparedness for future intervention scenarios.

It is also important to note that different countries had varying strategies regarding NPIs for SARS-CoV-2. Our study was conducted in a single city in Germany; therefore, our findings and conclusions might not be applicable to other regions. Further research in primary care practices is therefore critical to determine whether the high occurrence of viral co-detection reflects a rebound in respiratory virus activity following the relaxation of COVID-19-related NPIs or merely represents the typical spectrum of viral exposure among young children in community settings [32]. The high number of co-detection observed in our cohort, which was conducted in the later phase of the pandemic, may in part be explained by an overall increase in viral circulation during that period. Notably, despite frequent co-detection, clinical illness severity was not increased, suggesting that some of the detected viruses may be bystanders rather than causative agents.

These findings have practical implications for patient management, as they highlight that multiple virus detections do not necessarily call for more intensive treatment approaches. In addition, the results are relevant for understanding the pathogenesis of acute respiratory infections (ARI), where co-infections may not always result in additive or synergistic increases in clinical disease severity.

Taken together, our data emphasize the need for careful interpretation of multiplex PCR results in clinical decision-making, particularly in pediatric outpatient care. Future studies should aim to distinguish clinically relevant infections from incidental viral detections-for example, through sequential testing in children-in order to improve diagnosis, treatment, and public health strategies.

## Supplementary Information

Below is the link to the electronic supplementary material.

Supplementary Material 1

## Data Availability

The datasets generated and/or analysed during the current study are not publicly available due data protection regulations but are available from the corresponding author on reasonable request.

## Abbreviations

AdV: Adenovirus
ARI: Acute respiratory infections
EV: Enterovirus
hBoV: Human bocavirus
hMPV: Human metapneumovirus
hPeV: Human parechovirus
HPIV: Human parainfluenza virus 1–4
HRV: Human rhinovirus
IQR: Interquartile range
LRTI: Lower respiratory infection
N: Number
NPI: Non-pharmaceutical COVID-19 interventions
PCR: Polymerase chain reaction
RSV: Respiratory syncytial virus
URTI: Upper respiratory infection
