# Supplementary material for: High rates of viral co-detection in outpatient children with acute respiratory infections after the easing of preventive measures against Covid-19 in Germany
Source: BMC Infect Dis. 2026 Mar 24;26:740. doi: 10.1186/s12879-026-13125-9 (PMC13064178; doi:10.1186/s12879-026-13125-9)
Supplement: Supplementary file 1 — Supplementary Material 1 [file 12879_2026_13125_MOESM1_ESM.docx]

**Questionnaire CoPraKid 2.0**
University Children’s Hospital Würzburg
A. Haufschild, Dr. G. Engels, Prof. Dr. J. Liese

**1. Patient Data**

1.1 Sex: male ☐ female ☐ diverse ☐
1.2 Date of birth: (month-year): ____ - ____
1.3 Today’s date / date of consultation (day/month/year): ____ / ____ / ____
1.4 Symptom onset (day/month/year): ____ / ____ / ____
1.5 Hospital admission on day of consultation: yes ☐ no ☐

**2.1 Current symptoms**

(Check all that apply):
☐ Fever (≥ 38°C)
☐ Rhinitis
☐ Cough
☐ Nausea
☐ Abdominal pain
☐ Diarrhea
☐ Tachypnea
☐ Poor fluid intake
☐ Rash
☐ Tachycardia
☐ Hypoxia
☐ Dyspnea
☐ Headache
☐ Sore throat
☐ Others: ___________

2**.2 Current diagnoses**

(Check all that apply):
☐ Febrile infection
☐ Pharyngitis
☐ Tonsillitis
☐ Otitis media
☐ Pseudo/False croup
☐ Bronchitis / Bronchiolitis
☐ Pneumonia
☐ Respiratory tract infection
☐ Gastroenteritis
☐ Unclear/Unknown

**3. Underlying Conditions**

(Check all that apply):
☐ No underlying condition(s)
☐ Prematurity
☐ Asthma bronchiale / recurrent obstructive bronchitis
☐ Chronic lung disease (e.g. bronchopulmonary dysplasia)
☐ Congenital heart defect
☐ Congenital immunodeficiency or immunosuppression
☐ Oncological disease
☐ Nephrological disease
☐ Neurological disease
☐ Others: ___________

**4. Laboratory Results**

(This part of the questionnaire is completed after receiving the laboratory results)

4.1 Multiplex PCR: positive ☐ negative ☐
4.2 SARS-CoV-2: positive ☐ negative ☐
4.3 If Multiplex PCR positive, which viruses were detected? (Check all that apply):
☐ Rhinovirus
☐ Adenovirus
☐ Coronavirus NL63
☐ Coronavirus 229E
☐ Coronavirus OC43
☐ Coronavirus HKU1
☐ Influenza A
☐ Influenza A(H1N1)
☐ Influenza B
☐ Parainfluenza 1
☐ Parainfluenza 2
☐ Parainfluenza 3
☐ Parainfluenza 4
☐ Human metapneumovirus A
☐ Human metapneumovirus B
☐ RSV A
☐ RSV B
☐ Enterovirus
☐ Parechovirus
☐ Bocavirus
